# Silencing eL31 suppresses the progression of colorectal cancer via targeting DEPDC1

**DOI:** 10.1186/s12967-022-03663-6

**Published:** 2022-10-29

**Authors:** Gaowa Sharen, Xiongfeng Li, Jiaxin Sun, Lei Zhang, Wen Xi, Xiaodong Zhao, Fei Han, Longlong Jia, Rong A, Haidong Cheng, Mingxing Hou

**Affiliations:** 1 Department of Pathology, Affiliated Hospital of Inner Mongolia Medical University & Department of Pathological Anatomy, College of Basic Medicine of Inner Mongolia Medical University, Hohhot, 010059 Inner Mongolia China; 2 Graduate School , Inner Mongolia Medical University, Hohhot, 010063 Inner Mongolia China; 3 Department of Clinical Medicine Research Center, Affiliated Hospital of Inner Mongolia Medical University, Hohhot, 010059 Inner Mongolia China; 4 Department of Gastrointestinal Surgery, Affiliated Hospital of Inner Mongolia Medical University, Hohhot, 010059 Inner Mongolia China

**Keywords:** Colorectal cancer, eL31, DEPDC1

## Abstract

**Background:**

Colorectal cancer (CRC) is one of the most commonly diagnosed human malignancies. Ribosomal protein L31 (RPL31, aka eL31) is a component of the 60S large ribosomal subunit, and its expression pattern and functional role in CRC have not been reported.

**Methods:**

Herein, we identified that eL31 protein level was dramatically increased in CRC tissues through using IHC analysis. More notably, elevated eL31 was associated with larger tumor size and shorter overall survival. Besides, we evaluated the effects of eL31 depletion on CRC cell phenotypes in vitro.

**Results:**

The data indicated that eL31 knockdown restricted CRC cell proliferation, migration and colony formation whilst enhancing cell apoptosis. Importantly, eL31 was also essential for CRC tumor growth in vivo, as demonstrated by impaired tumor growth markers and reduced Ki67 levels in xenografts from eL31-depleted cells. In addition, our evidence indicated that DEP domain containing 1 (DEPDC1) was a potential downstream target of eL31 in regulating CRC. Consistently, DEPDC1 depletion restrained CRC cell proliferation and migration, as well as facilitated cell apoptosis. More interestingly, DEPDC1 depletion could reverse the promotion effects of eL31 elevation on CRC cells.

**Conclusions:**

Identification of eL31’s function in CRC may pave the way for future development of more specific and more effective targeted therapy strategies against CRC.

**Supplementary Information:**

The online version contains supplementary material available at 10.1186/s12967-022-03663-6.

## Background

Colorectal cancer (CRC) is recognized as the third highest cancer type in terms of incidence rate and the third most frequent cause of cancer death in both genders [1–3]. Increasingly, CRC accounts for approximately 10% of all new cancer cases globally [4, 5]. The current first-line treatments for CRC include surgical resection, chemotherapy, radiotherapy and targeted therapy [6, 7]. Although various treatment approaches have shown obvious anticancer activity in CRC and improved the survival rate among cancer patients, the majority of patients have poor prognosis and cannot be fully cured due to inevitable recurrence [8, 9]. Therefore, there is an urgent need to focus on improving the effectiveness and sensitivity of anticancer regimens, while an understanding of CRC pathogenesis and novel therapeutic targets may contribute to better selection of treatment approaches and reduced recurrence.

Growing research suggests that high levels of ribosomal protein expression are prognostic factors in certain tumors [10–13]. In addition, tumor cells are characterized by a higher production of ribosomes, necessary to maintain enhanced growth and subsequent cell division. The increase in ribosome production is closely related to dysregulated ribosome biogenesis and alteration in hallmarks of cancer cells such as nucleoli number, size and shape [14, 15].

The ribosome is a structurally and functionally conserved supramolecular ribonucleoprotein (RNP) complex, and its biogenesis is an energy-consuming and well-orchestrated process, requiring several assembly and maturation factors [16]. It is well known that ribosomal proteins are synthesized in the cytoplasm, and subsequently combine with rRNA in nucleolus to provide a supply of ribosomal subunits [17, 18]. In eukaryotic cells, the cytosolic 80S ribosome is composed of two subunits: the small 40S subunit and the large 60S subunit [16]. eL31, a protein that is part of the 60S large ribosomal subunit, was demonstrated to be one of the constituent proteins of the ribosomal P-site [19]. It is located at or near the vicinity of the peptidyl site of ribosomes, is an important constituent of peptidyltransferase center, and belongs to the eukaryotic ribosomal proteins [20]. Furthermore, eL31 forms a rim around the polypeptide tunnel exit, which physically interacts with Zuo1 subunit of RAC (ribosome-associated complex) [21]. Several lines of evidence recently presented that eL31 was involved in the proliferation of bicalutamide-resistant prostate cancer cells [22, 23]. In addition to these, eL31 was recently identified as a novel Diamond Blackfan anemia (DBA) gene [24]. On the other hand, HL31, which is highly homologous to eL31, has increased expression in colorectal tumors [25]. However, the mechanistic role of eL31 in CRC remains poorly understood and warrants further investigation.

Here, we set out to characterize the function and mechanism of action of eL31 in CRC development in more detail. To that end, IHC analysis was employed to identify abundant eL31 expression in CRC tissues, which was associated with tumor size and prognosis of patients. Moreover, we found that eL31 depletion resulted in the restrain of cell growth and the promotion of cell apoptosis. Besides, the findings of in vivo experiments was consistent with the above results, indicating an inhibitory effect of eL31 depletion on CRC tumorigenesis. Additionally, the investigation on the regulatory mechanism of eL31 in CRC identified DEPDC1 as a potential downstream target of eL31. In summary, eL31 functions as tumor promotor in CRC, which may represent a novel therapeutic target for CRC.

## Methods

### Tissue specimens and cell lines

A tissue microarray (Cat. HColA180Su17, n = 101 CRC tissues and n = 77 morphological normal tissues) was provided by Shanghai Outdo Biotech Company. This study was approved by the Institutional Animal Care and Use Committee of Affiliated Hospital of Inner Mongolia Medical University and the Ethics committee of Affiliated Hospital of Inner Mongolia Medical University.

Human normal colorectal mucosal cell FHC and a panel of CRC cell lines including HCT 116, RKO and HT29 were obtained from Cell Resource Center, Institute of Basic Medicine, Chinese Academy of Medical Sciences (Beijing, China). The culture and treatments were performed as follows: HT29 cells were cultured in McCoy′s5A + 10%FBS medium, and the other three types of cells were cultured in 1640 medium with 10% FBS, placing in a 37 °C incubator containing 5% CO_2_.

### Immunohistochemistry (IHC)

The tissue specimens were soaked with xylene and washed with alcohol (China National Pharmaceutical Group Co., Ltd, Beijing, China). After that, the slides were repaired with 1 × EDTA (Beyotime Biotechnology Co., Ltd, Shanghai, China) and blocked with 3% H_2_O_2_ and serum. Next, primary antibodies or secondary antibodies were used to incubate slides at 4 °C overnight, and then the slides were stained using DAB and hematoxylin (Baso DiagnosticsInc., Zhuhai, China). Finally, the slides were sealed with neutral resin (China National Pharmaceutical Group Co., Ltd, Beijing, China). The images were scored as negative (0), positive (1–4), +  + positive (5–8), or +  +  + positive (9–12), based on the sum of the staining intensity (varied from weak to strong) and staining extent scores, which graded as 0 (0%), 1 (1–25%), 2 (26–50%), 3 (51–75%), or 4 (76–100%). Antibodies used were listed in Additional file 1: Table S1.

### The Cancer Genome Atlas (TCGA) and GenomicScape database analysis

In this study, our expression profile analysis was based on the RNAseq counts data of 471 CRC and 41 normal tissues from The Cancer Genome Atlas website (TCGA, https://tcga-data.nci.nih.gov/tcga/). In addition, we analyzed 76 samples with low eL31 and 101 samples with high eL31 from the GenomicScape website (http://www.genomicscape.com) to investigate the relationship between patient overall survival and eL31 levels.

### Plasmid construction and lentivirus transfection

eL31 and DEPDC1 RNAi/overexpression target sequences were designed by Shanghai Bioscienceres Co., Ltd. (Shanghai, China), subsequently inserted into the BR-V-108 vector through the restriction sites at both ends and transformed into TOP 10 E. coli competent cells (Tiangen, Beijing, China). The plasmids of positive recombinants were extracted with the EndoFree maxi plasmid kit (Tiangen, Beijing, China), and the concentration of which was determined in a spectrophotometer (Thermo_Nanodrop 2000). RKO or HCT-116 cells in logarithmic growth phase were transfected by adding 40 μL 1 × 10^8^ TU/mL lentivirus, culturing in 1640 medium with 10% FBS in a 6-well dish (2 × 10^5^ cells/well). The cell transfection efficiency and knockdown efficiency were evaluated.

### RNA extraction, cDNA synthesis and qRT-PCR

Total RNAs were extracted according to the standard methods of TRIzol reagent (Sigma, St. Louis, MO, USA). cDNA synthesis and qRT-PCR were performed using Promega M-MLV Kit (Promega Corporation, Madison, Wisconsin, USA) and the SYBR Green Mastermixs Kit (Vazyme, Nanjing, Jiangsu, China). The mRNA level of GAPDH was used as an internal normalization control. The relative expression of mRNA was calculated by the 2^−△△Ct^ method. The primers sequences (5′-3′) were listed in Additional file 1: Table S2.

### Western blot assay and Co-immunoprecipitation (Co-IP)

After transfection, RKO and HCT-116 were harvested and lysed with 1 × Lysis Buffer lysis (Cell Signal Technology, Danvers, MA), and quantified with BCA methods. At the same time, 10% SDS-PAGE was used to segregate total proteins and transferred into PVDF membranes, followed by blocking in TBST solution containing 5% non-fat milk and subsequently incubating with primary antibodies at room temperature for 1 h. Next, the membranes were incubated with secondary antibodies and washed with TBST solution for three times (10 min/time). Finally, the ECL + plusTM western blotting system kit was used for color rendering, and X-ray imaging was captured.

In Co-IP analysis, HCT 116 cells were lysed, and total proteins were collected. Then, 1.0–1.2 mg proteins were incubated with antibody overnight, followed by 2 h of incubation with 20 μL beads at 4 °C. After that, the cleared protein antibody beads complex was incubated at 95–100 °C for 10 min. Then the proteins in immunocomplex were separated by 10% SDS-PAGE as western blot assay. Finally, primary and secondary antibodies were added to identify interacting proteins. Antibodies used in western blot assay showed in Additional file 1: Table S1.

### Cell proliferation detection

For MTT assay, RKO or HCT-116 cells were harvested, digested and resuspended into the cell suspension. 100 μL cell suspension was cultured in 96-well plates at the cell density of 2000 cells and determined for 5 days. 20 μL MTT (5 mg/mL) and 100 μL DMSO were added into 96-well plates. Optical density (OD) value at 490 nm was detected with microplate reader (Tecan infinite, Mannedorf Zurich, Switzerland).

For Celigo cell counting assay, RKO or HCT 116 cells were collected after transfecting indicated lentiviruses. The cells were cultured until the fusion reached 70—90%. Then the cells were seeded into 96-well plates at the density of 2,000 cells/well, placing in an incubator with 5% CO_2_ at 37 °C. The cell images were taken by Celigo image cytometer (Nexcelom Bioscience, Lawrence, MA, USA) and a continuous 5-day cell proliferation curve was drawn.

### Colony formation assay

HCT 116 or RKO cells with indicated lentiviruses were digested and resuspended to seed into a 6-well plate (2 mL/well). Then the cells were cultured for 8 days and the medium was changed every 3 days to form colony. Visible clones in 6-well plate were recorded by fluorescence microscope (Olympus, Tokyo, Japan). Finally, the cells were washed with PBS, fixed with 1 mL 4% paraformaldehyde and stained by 500 μL Giemsa (Dingguo, Shanghai, China) and the number of colonies was recorded.

### Cell migration assay

Wound-healing assay was performed to evaluate cell migration ability. RKO and HCT-116 cells, transfected with indicated lentiviruses, were seeded into a 96-well plate (5 × 10^4^ cells/well). Then, the cells were incubated in an incubator with 5% CO_2_ at 37 °C. The cells were observed in a microscope at 24 h and 64 h. The experiment was repeated 3 times and the migration rate of cells was evaluated based on the scratch images.

For transwell assay, RKO and HCT-116 cells with indicated lentiviruses were cultured to reach the density of 1 × 10^5^ cells/mL and loaded into the upper chamber containing serum-free medium. Then, the upper chamber was transferred to the lower chamber with 30% FBS and incubated for 72 h. Finally, 400 µL Giemsa was added for cell staining, and the cell migration ability was quantified.

### Flow cytometry assay for cell apoptosis

Lentivirus-transfected RKO and HCT-116 cells were cultured in 6-well plates (2 mL/well) for 5 days. 10 μL Annexin V-APC was added for 10–15 min at room temperature in the dark. The cell apoptosis level was measured by using FACSCalibur (BD Biosciences, San Jose, CA, USA).

### Human apoptosis antibody array

The effects of eL31 loss on apoptosis-related protein expression in HCT 116 cells were detected by the Human Apoptosis Antibody Array. After the cells were lysed, Handling Array membranes were blocked in 2 mL 1 × Wash Buffer II and incubated with cell lysates and 1 × Biotin-conjugated Anti-Cytokines overnight at 4 °C. Finally, the signals of membranes were tracked by chemiluminescence imaging system.

### PrimeView human gene expression array

Total RNAs were extracted using the RNeasy kit (Sigma, St. Louis, MO, USA). The quality and integrity of obtained RNA were determined by Nanodrop 2000 (Thremo Fisher Scientific, Waltham, MA, USA) and Agilent 2100 and Agilent RNA 6000 Nano Kit (Agilent, Santa Clara, CA, USA). According to the manufacturer’s instruction, we performed an Affymetrix human GeneChip PrimeView, and the outcomes were scanned by Affymetrix Scanner 3000 (Affymetrix, Santa Clara, CA, USA). The statistical significance of raw data was completed by using a Welch t-test with Benjamini–Hochberg FDR (|Fold Change|≥ 1.3 and *FDR* < 0.05 as significant). Functional enrichment analysis and interactive network analysis based on Ingenuity Pathway Analysis (IPA) (Qiagen, Hilden, Germany) were executed, and |Z—score|> 0 is considered valuable.

### The construction of nude mouse tumor formation model

All animal experiments conformed to the European Parliament Directive (2010/63/EU) and were approved by the Institutional Animal Care and Use Committee of Affiliated Hospital of Inner Mongolia Medical University and the Ethics committee of Affiliated Hospital of Inner Mongolia Medical University. Four-week-old female BALB-c nude mice (Shanghai Weitong Lihua Animal Research Co., Ltd.) were used to establish a xenograft model. The mice were randomly divided into two groups (n = 5/group), and 4 × 10^6^ well-grown HCT 116 cells with sheL31 and shCtrl were suspended in 100 μL of PBS and subcutaneously injected subcutaneously into nude mice. The tumor volume was tested during the entire feeding period. After 38 days, the mice were euthanized and the tumors were removed to weigh and photograph, and finally frozen in liquid nitrogen and stored at − 80 °C.

### Statistical analysis

Statistical analysis was carried out using GraphPad Prism 8 (San Diego, CA, USA) and SPSS 19.0 (IBM, SPSS, Chicago, IL, USA). All data were represented as the mean ± SD from at least three repeated experiments. Student’s t-test (for comparisons of two groups) and one-way ANOVA (for multiple group comparisons) were used to analyze the statistical significance. The Spearman correlation analysis and Mann–Whitney U analysis were used to assess the association between eL31 expression and characteristics of CRC patients. *P* < 0.05 was considered to be significantly different.

## Results

### eL31 protein expression patterns in colorectal cancer tissues

**Fig. 1:**
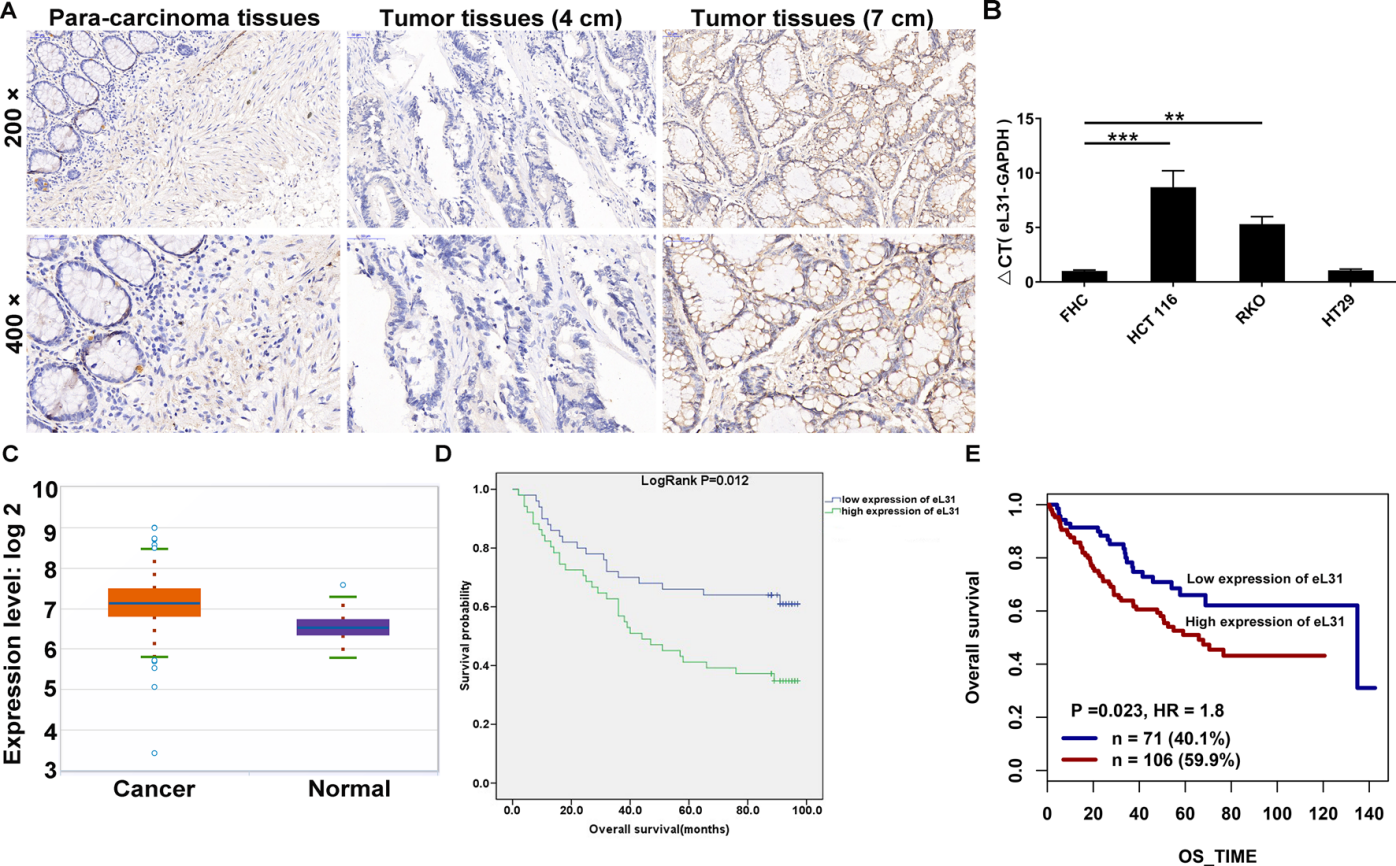
eL31 was up-regulated in colorectal cancer tissues. **A** The expression levels of eL31 in colorectal cancer tumor tissues and para-carcinoma tissues were determined by immunohistochemical staining. **B** The expression levels of eL31 mRNA was detected in colorectal cancer cells and normal colorectal mucosal cell FHC. **C** The eL31 expression data was assessed for 471 CRC and 41 normal tissues cases from The Cancer Genome Atlas website (TCGA). **D** Kaplan–Meier survival analysis was performed to reveal the relationship between eL31 expression and prognosis of colorectal cancer patients. **E** eL31 is an independent prognostic factor for CRC through analyzing GenomicScape website. Results were presented as mean ± SD. ** *P* < 0.01, *** *P* < 0.001

A tissue microarray including 101 cases of CRC tissues and 77 para-carcinoma tissue samples was employed. Using IHC detection, we found overexpressed eL31 in cancer tissues relative to para-carcinoma tissues (*P* < 0.001, Fig. 1A and Table 1). In addition, we detected eL31 mRNA levels in human normal colorectal mucosal cell FHC and a panel of CRC cell lines, obtaining abundant eL31 mRNA levels in CRC cell lines, especially in HCT 116 and RKO cells (Fig. 1B). Moreover, we analyzed eL31 expression in 471 CRC and 41 normal tissues cases from The Cancer Genome Atlas database (TCGA, https://tcga-data.nci.nih.gov/tcga/), finding that CRC tissues exhibited a higher level of eL31 compared with normal tissues (Fig. 1C). The correlation between eL31 levels and various clinicopathological features was then investigated. The results demonstrated that high eL31 expression appeared to be more prevalent in > 4 cm tumor tissues than in ≤ 4 cm tumor tissues, with the difference being statistically significant (*P* < 0.05, Fig. 1A and Table 2). Through the Spearman rank correlation analysis, eL31 level was again found to be significantly related to tumor size (*P* < 0.05, Table 3). Furthermore, we investigated the association between eL31 levels and patients’ overall survival using Kaplan–Meier analysis and the GenomicScape website (http://www.genomicscape.com), which both implied the prognostic potential of eL31 in CRC (Fig. 1D and E). These data indicated that eL31 was involved in CRC development and progression, and might function as a potential prognostic marker.

**Table 1:** Expression patterns of eL31 in colorectal cancer tissues and para-carcinoma tissues revealed in immunohistochemistry analysis

eL31 expression	Tumor tissue		Para-carcinoma tissue		*P* value
	Cases	Percentage	Cases	Percentage	
Low	50	49.5%	72	93.5%	< 0.001
High	51	50.5%	5	6.5%

**Table 2:** Relationship between eL31 expression and tumor characteristics in patients with colorectal cancer

Features	No. of patients	eL31 expression		*P* value
		low	high	
All patients	101	50	51	
Age (years)				0.766
< 69	51	26	25	
≥ 69	50	24	26	
Gender				0.488
Male	50	23	27	
Female	51	27	24	
Tumor size				0.026
≤ 4 cm	36	23	13	
> 4 cm	64	26	38	
Grade				0.374
I	1	1	0	
II	83	42	41	
III	16	6	10	
IV	1	1	0	
Positive lymph nodes				0.329
≤ 0	59	31	28	
> 0	40	17	23	
Stage				0.184
1	6	4	2	
2	55	29	26	
3	39	17	22	
4	1	0	1	
T Infiltrate				0.147
T1	1	1	0	
T2	5	3	2	
T3	75	39	36	
T4	19	7	12	
M value				0.332
M0	100	50	50	
M1	1	0	1	
Lymphatic metastasis (N)				0.192
N0	61	33	28	
N1	30	14	16	
N2	10	3	7

**Table 3:** Relationship between eL31 expression and tumor size in patients with colorectal cancer

		eL31
Tumor size	Spearman correlation	0.223
	Signification (double-tailed)	0.025
	N	100

### Loss of eL31 in tumor cells leads to decrease cell proliferation, migration and colony formation, increase apoptosis

**Fig. 2:**
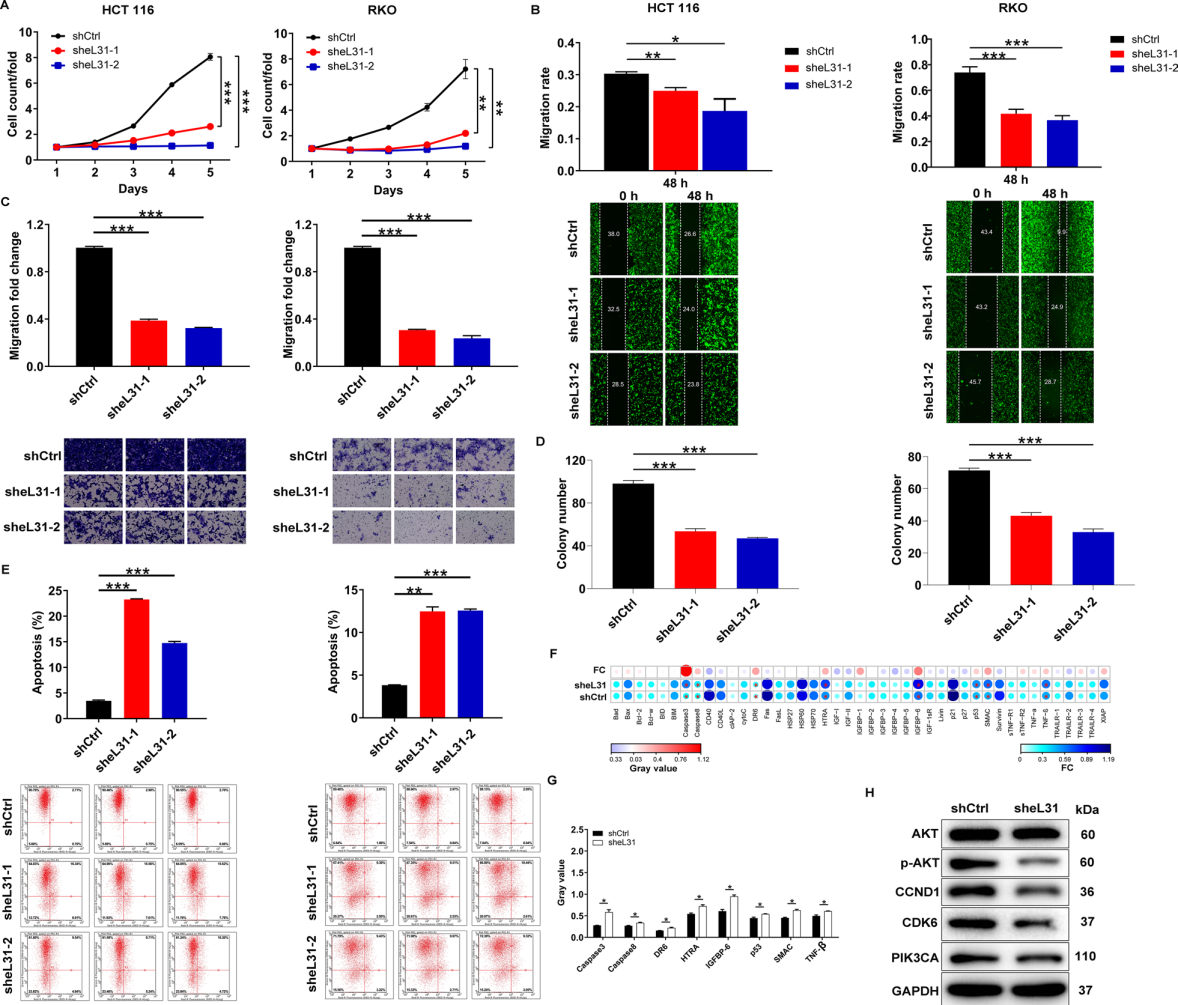
eL31 knockdown inhibited colorectal cancer development in vitro. **A** The cell proliferation rate was evaluated in colorectal cancer cell lines after transfection by MTT assay. **B** The migration rate of cells was detected in colorectal cancer cell lines after transfection by wound-healing assay. **C** The migration rate of cells was detected in colorectal cancer cell lines after transfection by transwell assay. **D** The effects of eL31 knockdown on colony formation were determined. **E** The effects of eL31 knockdown on cell apoptosis were examined by flow cytometry. **F** The expression of apoptosis-related proteins in HCT 116 cells transfected with sheL31 was measured by ECL with Human Apoptosis Antibody Array. The results circled in red indicated that the protein expression was up-regulated and *P* < 0.05. **G** Protein expression was presented in grayscale and visualized by R studio. Results were presented as mean ± SD. **H** The expression of Akt, P-Akt, CCND1, CDK6 and PIK3CA was detected by western blot. Magnification times: 200× . Results were presented as mean ± SD. * *P* < 0.05, ** *P* < 0.01, *** *P* < 0.001

In this section, we wondered whether loss of eL31 in CRC cells could induce changes in cell phenotypes. To address this, we transfected two lentivirus plasmids expressing eL31 (sheL31-1 and sheL31-2) into HCT 116 cells, which were employed for subsequent study due to their considerable knockdown efficiencies (*P* < 0.05, Additional file 1: Figure S1A). The results presented in Additional file 1: Figure S1B also reminded us the successful establishment of eL31-depleted HCT 116 and RKO cell models. Then, analysis of the cell proliferation revealed obviously slower proliferation rate in both cells after being transfected with sheL31-1 or sheL31-2 (*P* < 0.001, Fig. 2A). What’s more, we proposed that eL31 loss in tumor cells could exclusively restrain cell migration, corroborating our conjecture by wound-healing and transwell assays (Fig. 2B and C). Of note, we also demonstrated that, compared with shCtrl-transfected cells, sheL31-transfected cells displayed attenuated colony formation and increased apoptosis (*P* < 0.001, Fig. 2D and E). Also, the results of Human Apoptosis Antibody Array explained that increased cell apoptosis was induced by the overexpression of pro-apoptotic elements such as Caspase3, Caspase8, DR6, HTRA, IGFBP-6, p53, SMAC and TNF-β (Fig. 2F and G). Simultaneously, the downregulation of P-Akt, CCND1, CDK6 and PIK3CA expression was observed in HCT 116 cells upon silencing eL31 (Fig. 2H and Additional file 1: Figure S1C), through which eL31 knockdown might mediate cell growth and metastasis. Together, these results demonstrated that loss of eL31 in CRC cells induced a decrease in proliferation, migration and colony formation, an increase in apoptosis.

### Loss of eL31 leads to impair CRC tumorigenesis

**Fig. 3:**
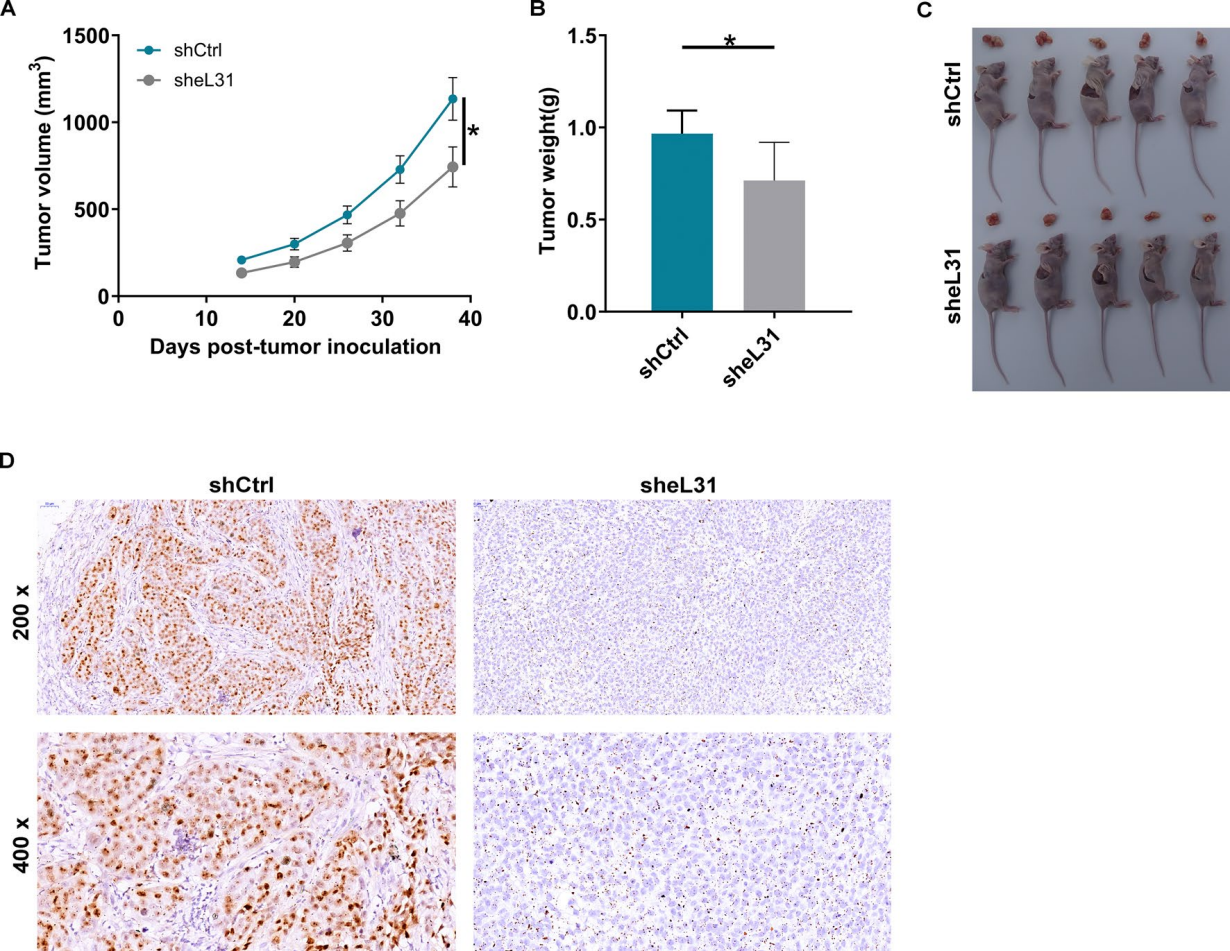
eL31 knockdown inhibited colorectal cancer tumor growth in vivo. **A** The volume of tumors was tested from feeding to sacrifice. **B** The weight of tumors was measured after sacrificing mice. **C** The photograph of tumors was taken after removing tumors. **D** The value of Ki-67 was detected by IHC in tumor sections. Magnification times: 200× . Results were presented as mean ± SD. * *P* < 0.05

The above results motivated us to study the effects of eL31 loss on CRC in vivo. For validation, xenografted models were established through subcutaneous injection of shCtrl or sheL31-transfected HCT 116 cells into four-week-old BALB/c nude mice. As shown in Fig. 3A and 3B, tumor characterization including volume and weight revealed a role for eL31 depletion in impairing tumorigenesis. 38 days later, mice were euthanized to harvest tumor (Fig. 3C). Finally, IHC staining confirmed the downregulation of proliferation marker Ki-67 expression in tumor tissues derived from eL31-depleted cells (Fig. 3D). Thus, depleting eL31 led to a significant inhibition in CRC tumorigenesis.

### eL31 targets DEPDC1 to regulate CRC

**Fig. 4:**
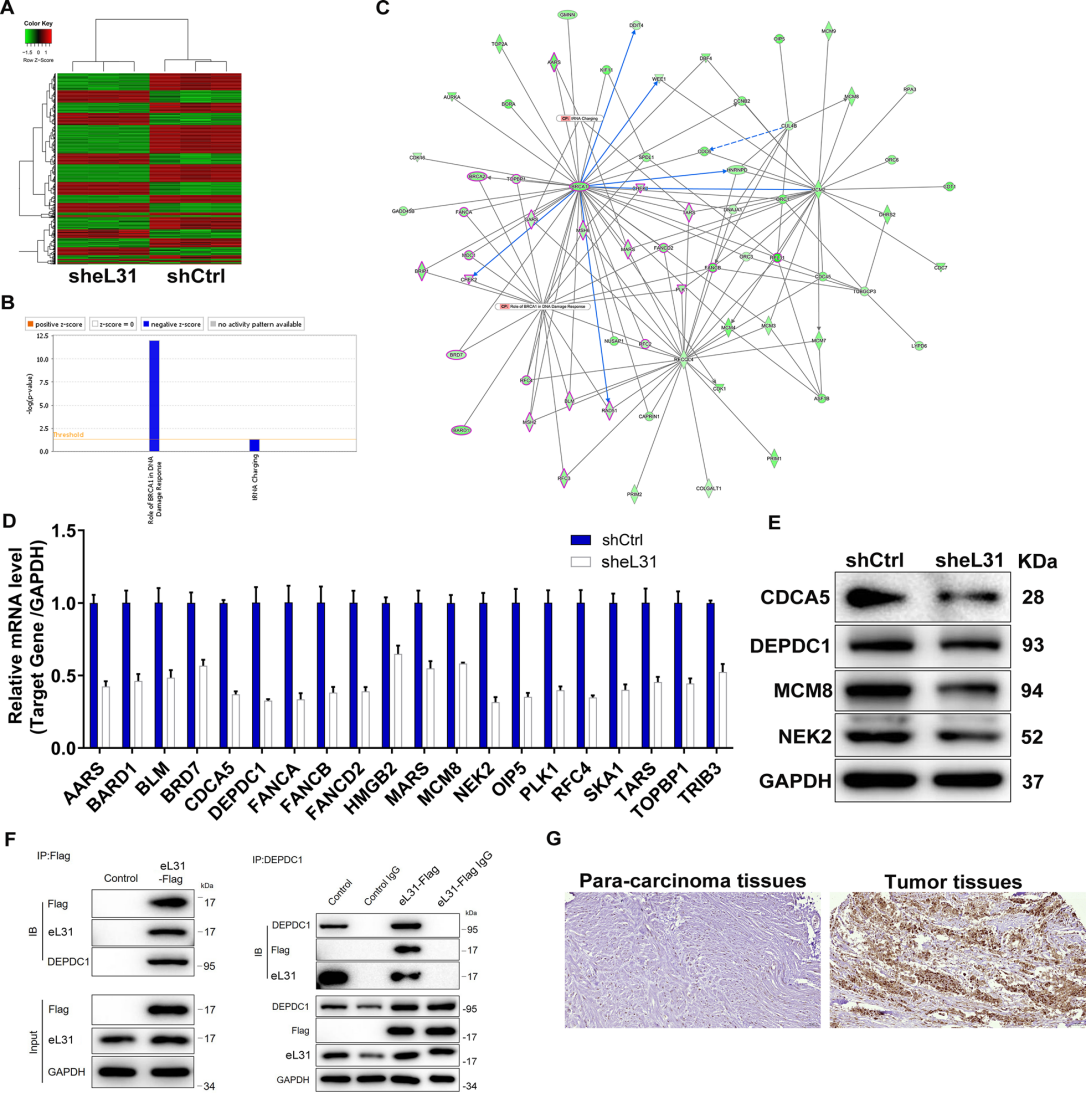
Exploration and verification of underlying mechanism of eL31 regulating colorectal cancer. **A** The heatmap of DEGs identified by PrimeView human gene expression array of HCT 116 cells treated with shCtrl (n = 3) or sheL31 (n = 3). **B** The enrichment of the DEGs in canonical signaling pathways was analyzed by IPA. **C** Interaction network diagram between DEGs was analyzed by IPA. **D**, **E** The expression of several DEGs were identified by qRT-PCR (**D**) and western blot (**E**) experiments in HCT 116 cells with sheL31. **F** Co-IP assay was used to verify whether there was protein interaction between eL31 and DEPDC1. **G** The expression of DEPDC1 in tissues of normal and tumor was detected by IHC analysis

In addition to the above, we explored the underlying mechanism of eL31 silencing to overcome CRC. PrimeView human gene expression array was performed in shCtrl or sheL31-transfected HCT 116 cells, and 1166 differentially expressed genes (DEGs), specifically, 486 were up-regulated and 680 were down-regulated in sheL31-transfected HCT 116 cells (Fig. 4A and Additional file 1: Figure S2A). Moreover, we found that most downregulated genes were enriched in pathways: Role of BRCA1 in DNA Damage Response and tRNA Charging, implying that both pathways were significantly restrained due to eL31 knockdown (Fig. 4B).Subsequently, the interactive network analysis based on IPA revealed the relationship between eL31 with the above pathways (Fig. 4C), indicating that eL31 could affect multiple genes in these pathways. Out of them, we selected 20 genes for qRT-PCR detection and 4 for western blot verification in eL31-depleted HCT 116 cells. As present in Fig. 4D, 4E and Additional file 1: Fig. S2B, the mRNA and protein levels CDCA5, DEPDC1, MCM8 and NEK2 were found to be apparently decreased. On the other hand, Co-IP analysis confirmed that DEPDC1 could endogenously interact with eL31 (Fig. 4F). Furthermore, in line with eL31’s expression in CRC, IHC analysis also revealed DEPDC1 upregulation in CRC tumor tissues (Fig. 4G). We thus concluded that DEPDC1 is a downstream target of eL31 regulating CRC.

### DEPDC1 depletion reverses the promotion effects of eL31 overexpression on CRC

**Fig. 5:**
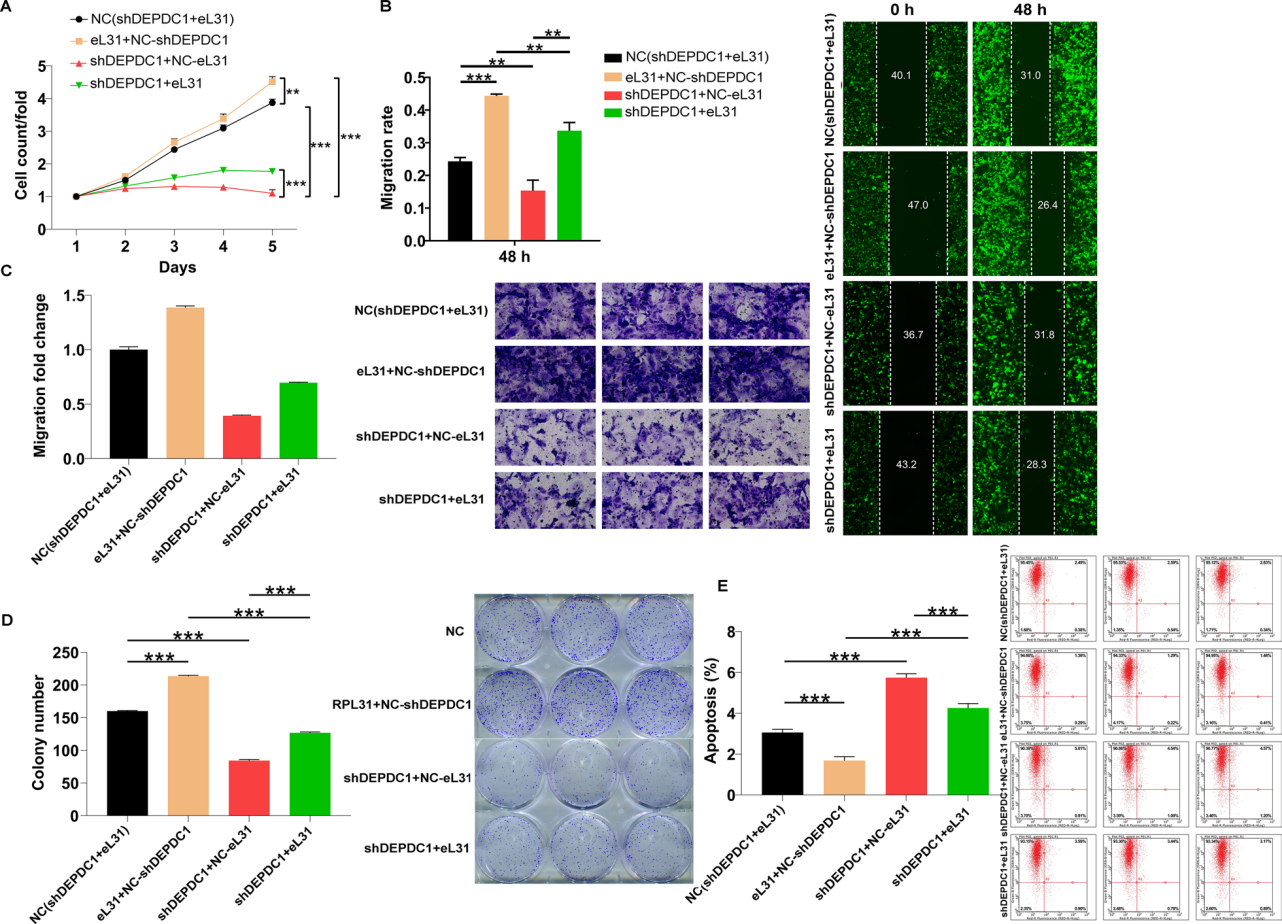
DEPDC1 depletion reversed the promotion effects of eL31 overexpression on CRC. **A** Celigo cell counting assay was employed to show the effects of eL31, shDEPDC1 and eL31 + shDEPDC1 on HCT 116 cell proliferation. **B**, **C** The migration rate of cells was detected in eL31, shDEPDC1 and eL31 + shDEPDC1 groups by wound-healing assay (**B**) and transwell assay (**C**). **D** Colony formation assay was used to evaluate the ability of HCT 116 cells to form colonies in eL31, shDEPDC1 and eL31 + shDEPDC1 groups. **E** The flow cytometry was performed to present the changes of HCT 116 cell apoptosis after transfecting eL31, shDEPDC1 and eL31 + shDEPDC1. NC (shDEPDC1 + eL31): Control; eL31 + NC-shDEPDC1: eL31 overexpression; shDEPDC1 + NC-eL31: DEPDC1 downregulation; shDEPDC1 + eL31: DEPDC1 downregulation and eL31 overexpression. The data are expressed as mean ± SD. ** *P* < 0.01, *** *P* < 0.001

Finally, the effects of the eL31-DEPDC1 axis on CRC cell behaviors were investigated, we constructed eL31-overexpressed, DEPDC1-silenced as well as DEPDC1-silenced combining with eL31-overexpressed HCT 116 cell models to perform recovery experiments (Additional file 1: Figure S3). The findings demonstrated that merely overexpressing eL31 enhanced cell proliferation, migration and colony formation, simultaneously attenuated apoptosis; the consumption of DEPDC1 alone obtained completely opposite phenotypes. Upon knocking down DEPDC1 in eL31-overexpressed cells, the promotion effects of eL31 overexpression on cell proliferation, migration and colony formation of CRC cells could be reversed (Fig. 5A-D), while the capacity of cell apoptosis was ameliorated (Fig. 5E).

On the other hand, we cultured RKO cells transfected with shDEPDC1 or shDEPDC1 + sheL31 and measured the transfection efficiency, as shown in Additional file 1: Figure S4A and S4B. Consistent with the above results, DEPDC1 depletion suppressed cell proliferation and migration, while facilitating cell apoptosis (*P* < 0.01, Additional file 1: Figure S5A-S5E). Moreover, previously impaired growth and migration abilities in eL31-depleted cells were further restrained by knockdown of DEPDC1, and previously enhanced apoptotic potential was further improved (*P* < 0.001, Additional file 1: Figure S5A-S5E). Collectively, these results demonstrated that DEPDC1 exhibited similar effects on the development of CRC as eL31, the knockdown of which could reverse the promotion effects of eL31 overexpression on CRC.

## Discussion

CRC is a common malignant tumor in the digestive department, with high morbidity and mortality, and is one of the major health threats to human beings [1, 3]. There are multiple strategies for the management and treatment of CRC, such as surgical management, radiotherapy and chemotherapy [7, 8]. The current main challenges of management and treatment of CRC are resistance to conventional agents and accompanying side-effects [26]. As such, targeted therapy has attracted more attention in order to reduce adverse effects and overcome resistance [27].

As part of the 60S large ribosomal subunit, an increasing body of evidence recently presented that eL31 is associated with cell cycle and modulate cancer development and progression [22, 28]. However, the anti-tumor effects of eL31 on CRC had not been investigated in the existed publications. In this study, we investigated whether eL31 was capable of participating in the development of CRC. For this purpose, this study found that eL31 was upregulated in CRC and was significantly related to tumor size and patient’s prognosis. Concerning the implication of eL31 in CRC, in vitro data indicated an inhibitory effect of eL31 knockdown on cell proliferation and migration, as well as a promotion effect on apoptosis. Also, the effects of eL31 depletion on CRC tumorigenesis were studied in xenografted models. These findings from in vivo experiments further designated the function of eL31 in CRC, which was illustrated by the measurement of tumor growth index. Previous study revealed that eL31 is overexpressed in prostate carcinomas. More intriguingly, the protein levels of tumor suppressor p53 and its targets, p21 and MDM2, were increased in prostate cancer cells with downregulated eL31. Moreover, the degradation of p53 protein was curbed after eL31 knockdown. The suppression in growth and cell cycle from eL31 knockdown was partially recovered upon p53 siRNA transfection [22]. From this, the author proposed that eL31 was linked to the development of prostate cancer via p53 pathway. We hypothesized that eL31 could use the above same mechanism in mediating CRC progression, which needs to be explored in depth in our further work. On the other hand, such kind of inhibition induced by eL31 depletion led to the downregulation of cancer-associated elements P-Akt, CCND1, CDK6 and PIK3CA. More importantly, we found that these alterations were related to DEPDC1 downregulation.

DEPDC1, a highly conserved 92-kDa protein, has been reported to be involved in multiple biological processes such as transcription regulation, cell mitosis, and apoptosis [29–32]. DEPDC1 is related to cell cycle, mainly expressed during cell interphase, and plays a critical role in cell division in metaphase [33–35]. In addition, DEPDC1 is linked to cell apoptosis, specifically, it could promote the NK-dependent degradation of BCL-2 family protein MCL1 to regulate microtubule-targeting chemotherapeutics, thereby inducing apoptosis [30]. Of note, DEPDC1 was found to be highly expressed in bladder cancer [36], hepatocellular carcinoma [37], and multiple myeloma [38], acting as a novel diagnostic marker or prognostic predictor, indicating that overexpression of DEPDC1 might play a role in the development and progression of cancers. It was worth mentioning that DEPDC1 was reported to be associated with CRC cell growth or apoptosis [39–41]. In this direction, the present study was aimed to investigate the synergistic effects of eL31 and DEPDC1 on CRC development. In this study, we reported abundant DEPDC1 expression in CRC tissues. It was also concluded that DEPDC1 depletion mitigated the promotion effects of eL31 elevation on CRC.

## Conclusion

In conclusion, our study highlighted that eL31 may work as a tumor promotor in CRC via targeting DEPDC1. Accordingly, the current idea was that eL31 might be used as a promising therapeutic target for CRC. In addition, the lack of in vivo data on eL31/DEPDC1 is a limitation in this study, and more studies are needed to support the promotion role of eL31/DEPDC1 in CRC. These knowledge will, in turn, provide a theoretical basis for new strategies for the diagnosis and treatment of CRC.

## Supplementary Information

**Additional file 1**: **Figure S1.** (A) The transfection efficiencies of sheL31-1 and sheL31-2 in HCT 116 cells were detected by qRT-PCR. (B) The transfection efficiencies of sheL31-1 and sheL31-2 in HCT 116 and RKO cells were evaluated through observing the fluorescence inside cells. Magnification times: 200×. (C) The densitometric analyses of Akt, P-Akt, CCND1, CDK6 and PIK3CA proteins expression. * *P* < 0.05, *** *P* < 0.001. **Figure S2.** (A) The volcano plot of gene expression profiling in HCT 116 cells with or without eL31 knockdown. Red dots represent upregulated DEGs, green dots represent downregulated DEGs. (B) The densitometric analyses of CDCA5, DEPDC1, MCM8 and NEK2 proteins expression. ** *P* < 0.01, *** *P* < 0.001. **Figure S3.** The transfection efficiencies of eL31, shDEPDC1 and eL31+ shDEPDC1 in HCT 116 cells were assessed through observing the fluorescence inside cells. Magnification times: 200×. NC (shDEPDC1+eL31): Control; eL31+NC-shDEPDC1: eL31 overexpression; shDEPDC1+NC-eL31: DEPDC1 downregulation; shDEPDC1+eL31: DEPDC1 downregulation and eL31 overexpression. **Figure S4.** (A) The transfection efficiencies of shDEPDC1-1, shDEPDC1-2 and shDEPDC1-3 in RKO cells were detected by qRT-PCR. (B) The transfection efficiencies of shDEPDC1-3 and sheL31+shDEPDC1 in RKO cells were evaluated through qRT-PCR detection. * *P* < 0.05, ** *P* < 0.01, *** *P* < 0.001. **Figure S5. **(A) Celigo cell counting assay was employed to show the synergistic effects of eL31 and DEPDC1 downregulation on RKO cell proliferation. (B) The flow cytometry was performed to show the synergistic effects of silencing eL31 and DEPDC1 on RKO cell apoptosis. (C) Colony formation assay was used to evaluate the ability of RKO cells to form colonies in shDEPDC1 and sheL31+ shDEPDC1 groups. (D, E) The migration rate of cells was detected in shDEPDC1 and sheL31+ shDEPDC1 groups by transwell assay (D) and wound-healing assay (E). shCtrl: Control; shDEPDC1: DEPDC1 downregulation; shDEPDC1+sheL31: DEPDC1 downregulation and eL31 downregulation. The data are expressed as mean ± SD. ** *P* < 0.01, *** *P *< 0.001. **Table S1.** Antibodies used in western blotting and IHC. **Table S2.** Primers used in qRT-PCR.

## Data Availability

All data generated was showed in this manuscript. The datasets used and/or analysed during the current study are available from the corresponding author on reasonable request.
