# Supplementary material for: Silencing eL31 suppresses the progression of colorectal cancer via targeting DEPDC1
Source: J Transl Med. 2022 Oct 29;20:493. doi: 10.1186/s12967-022-03663-6 (PMC9617412; doi:10.1186/s12967-022-03663-6)
Supplement: Supplementary file 1 — Additional file 1: Figure S1. (A) The transfection efficiencies of sheL31-1 and sheL31-2 in HCT 116 cells were detected by qRT-PCR. (B) The transfection efficiencies of sheL31-1 and sheL31-2 in HCT 116 and RKO cells were evaluated through observing the fluorescence inside cells. Magnification times: 200×. (C) The densitometric analyses of Akt, P-Akt, CCND1, CDK6 and PIK3CA proteins expression. * P < 0.05, *** P < 0.001. Figure S2. (A) The volcano plot of gene expression profiling in HCT 116 cells with or without eL31 knockdown. Red dots represent upregulated DEGs, green dots represent downregulated DEGs. (B) The densitometric analyses of CDCA5, DEPDC1, MCM8 and NEK2 proteins expression. ** P < 0.01, *** P < 0.001. Figure S3. The transfection efficiencies of eL31, shDEPDC1 and eL31+ shDEPDC1 in HCT 116 cells were assessed through observing the fluorescence inside cells. Magnification times: 200×. NC (shDEPDC1+eL31): Control; eL31+NC-shDEPDC1: eL31 overexpression; shDEPDC1+NC-eL31: DEPDC1 downregulation; shDEPDC1+eL31: DEPDC1 downregulation and eL31 overexpression. Figure S4. (A) The transfection efficiencies of shDEPDC1-1, shDEPDC1-2 and shDEPDC1-3 in RKO cells were detected by qRT-PCR. (B) The transfection efficiencies of shDEPDC1-3 and sheL31+shDEPDC1 in RKO cells were evaluated through qRT-PCR detection. * P < 0.05, ** P < 0.01, *** P < 0.001. Figure S5. (A) Celigo cell counting assay was employed to show the synergistic effects of eL31 and DEPDC1 downregulation on RKO cell proliferation. (B) The flow cytometry was performed to show the synergistic effects of silencing eL31 and DEPDC1 on RKO cell apoptosis. (C) Colony formation assay was used to evaluate the ability of RKO cells to form colonies in shDEPDC1 and sheL31+ shDEPDC1 groups. (D, E) The migration rate of cells was detected in shDEPDC1 and sheL31+ shDEPDC1 groups by transwell assay (D) and wound-healing assay (E). shCtrl: Control; shDEPDC1: DEPDC1 downregulation; shDEPDC1+sheL31: DEPDC1 downregulation and [file 12967_2022_3663_MOESM1_ESM.docx]

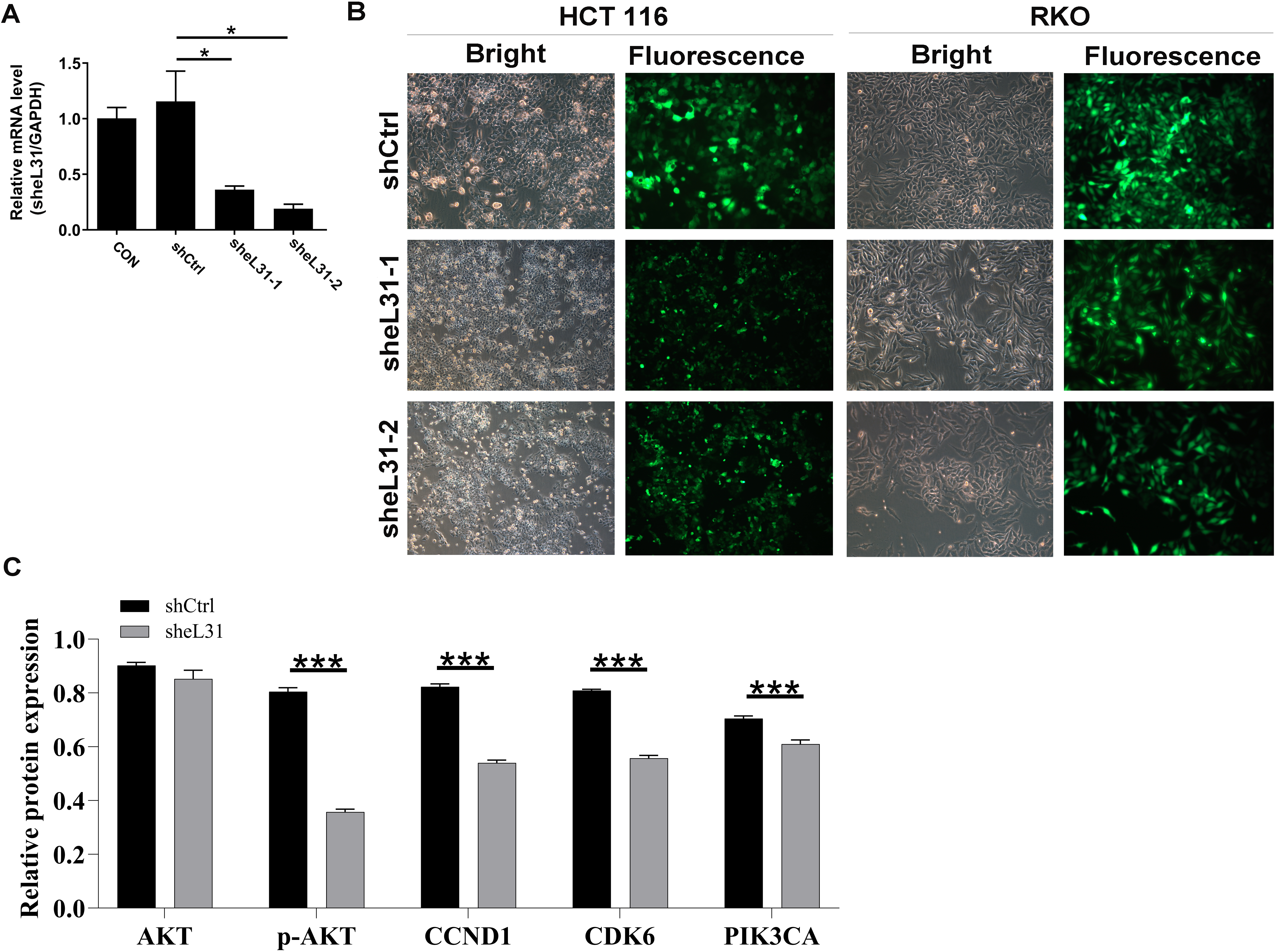


**Figure S1.** (A) The transfection efficiencies of sheL31-1 and sheL31-2 in HCT 116 cells were detected by qRT-PCR. (B) The transfection efficiencies of sheL31-1 and sheL31-2 in HCT 116 and RKO cells were evaluated through observing the fluorescence inside cells. Magniﬁcation times: 200×. (C) The densitometric analyses of Akt, P-Akt, CCND1, CDK6 and PIK3CA proteins expression. * *P* < 0.05, *** *P* < 0.001


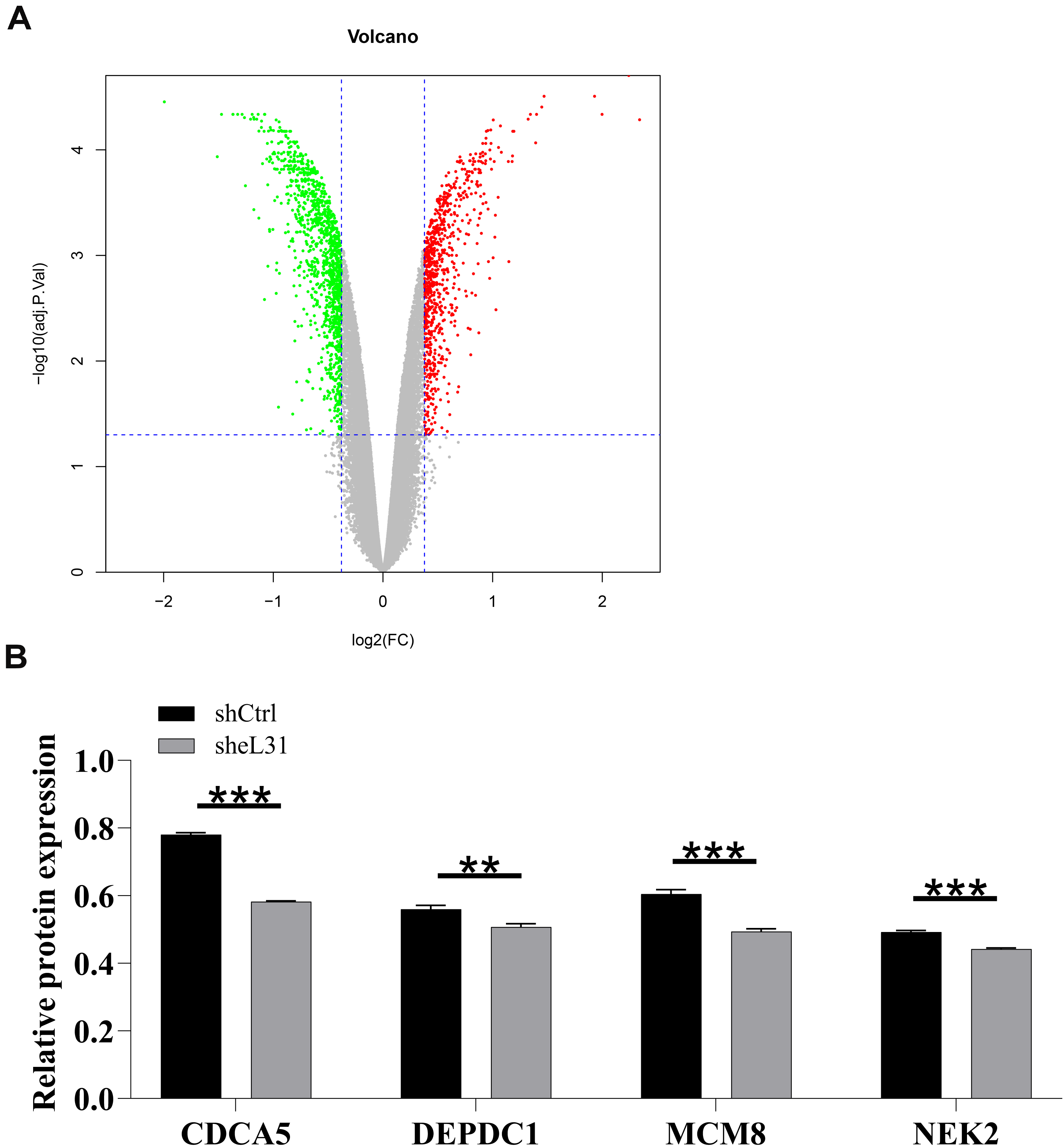


**Figure S2.** (A) The volcano plot of gene expression profiling in HCT 116 cells with or without eL31 knockdown. Red dots represent upregulated DEGs, green dots represent downregulated DEGs. (B) The densitometric analyses of CDCA5, DEPDC1, MCM8 and NEK2 proteins expression. ** *P* < 0.01, *** *P* < 0.001

**Figure S3.** The transfection efficiencies of eL31, shDEPDC1 and eL31+ shDEPDC1 in HCT 116 cells were assessed through observing the fluorescence inside cells. Magniﬁcation times: 200×. NC (shDEPDC1+eL31): Control; eL31+NC-shDEPDC1: eL31 overexpression; shDEPDC1+NC-eL31: DEPDC1 downregulation; shDEPDC1+eL31: DEPDC1 downregulation and eL31 overexpression.


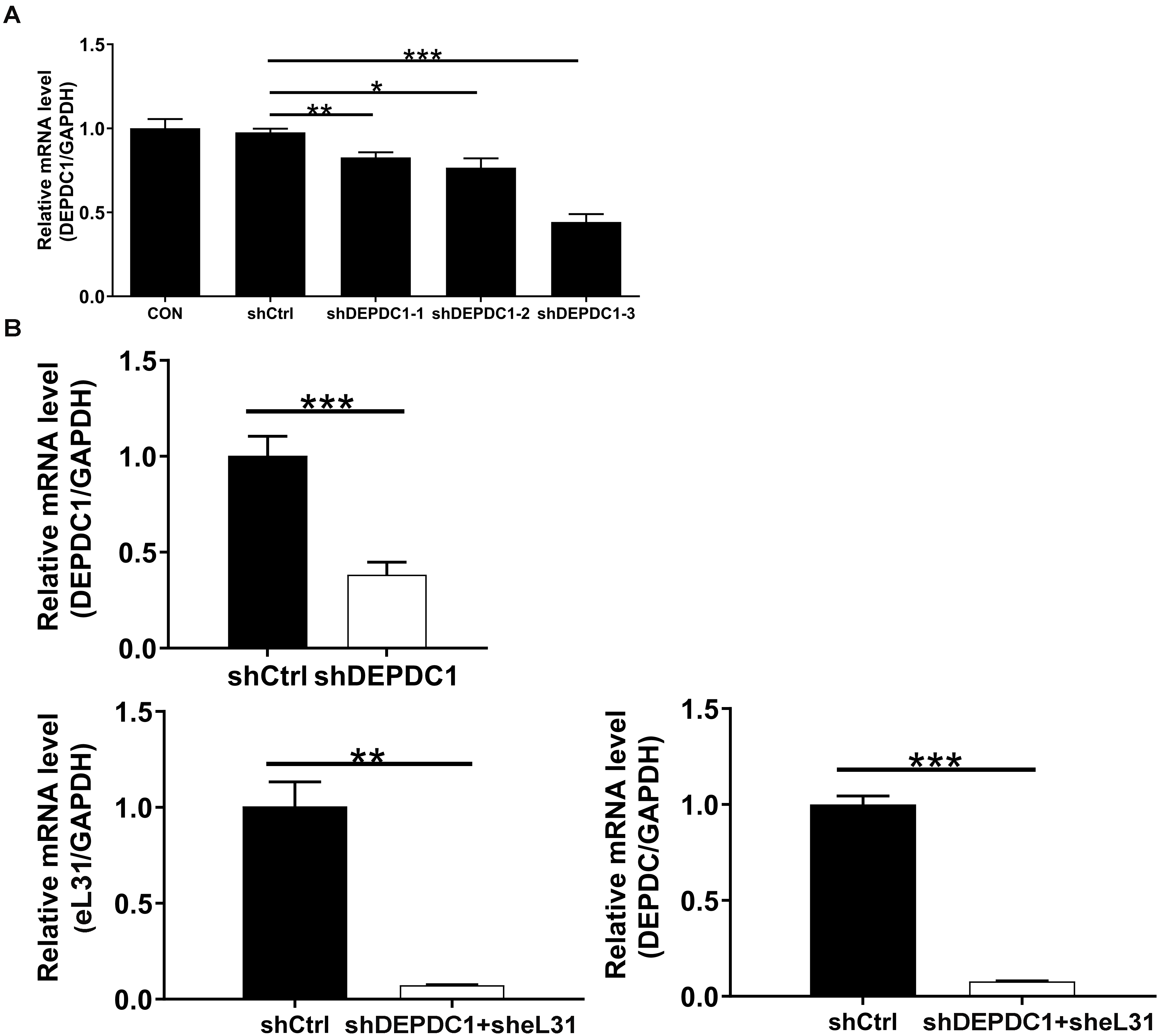


**Figure S4.** (A) The transfection efficiencies of shDEPDC1-1, shDEPDC1-2 and shDEPDC1-3 in RKO cells were detected by qRT-PCR. (B) The transfection efficiencies of shDEPDC1-3 and sheL31+shDEPDC1 in RKO cells were evaluated through qRT-PCR detection. * *P* < 0.05, ** *P* < 0.01, *** *P* < 0.001


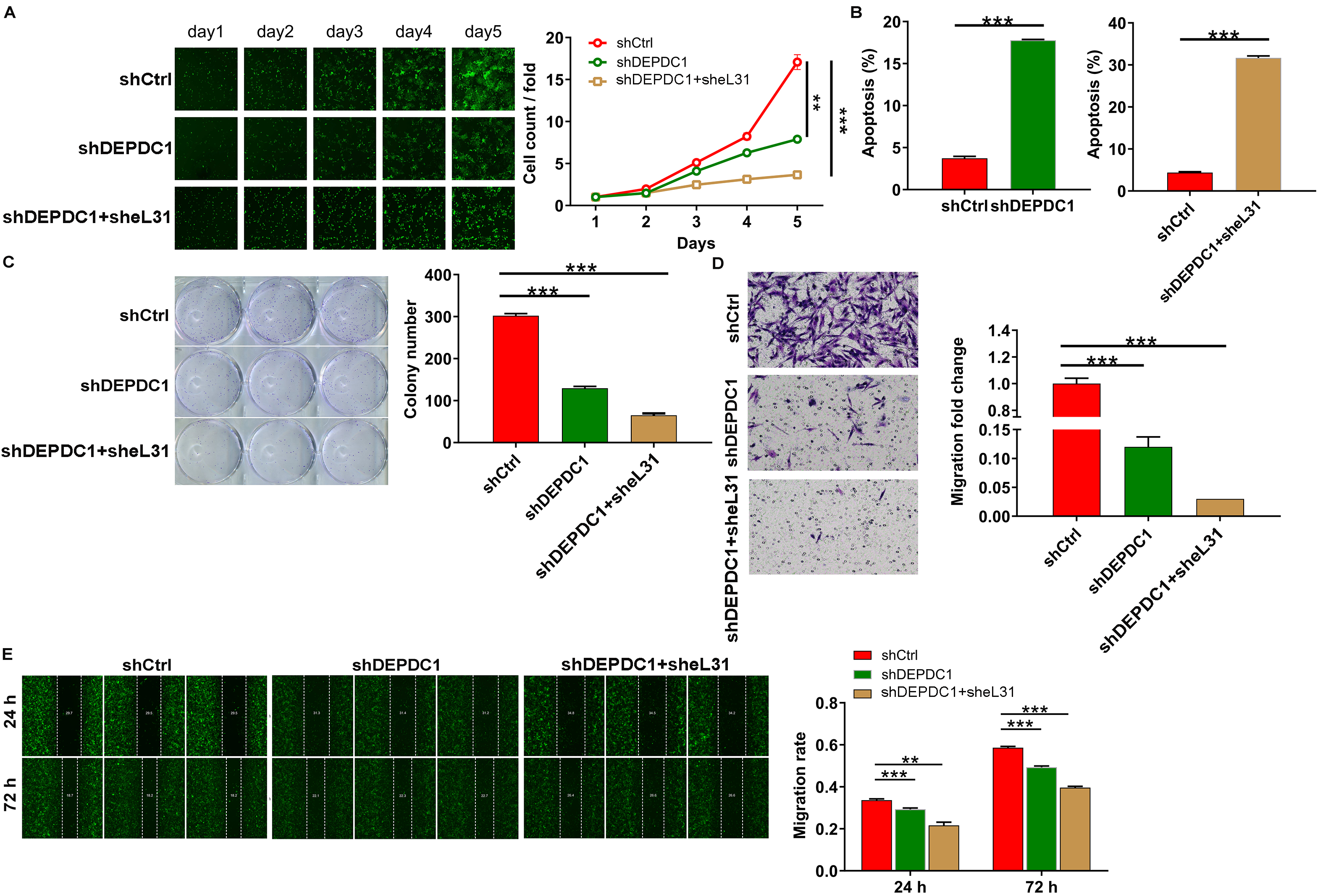


**Figure S5.** (A) Celigo cell counting assay was employed to show the synergistic effects of eL31 and DEPDC1 downregulation on RKO cell proliferation. (B) The flow cytometry was performed to show the synergistic effects of silencing eL31 and DEPDC1 on RKO cell apoptosis. (C) Colony formation assay was used to evaluate the ability of RKO cells to form colonies in shDEPDC1 and sheL31+ shDEPDC1 groups. (D, E) The migration rate of cells was detected in shDEPDC1 and sheL31+ shDEPDC1 groups by transwell assay (D) and wound-healing assay (E). shCtrl: Control; shDEPDC1: DEPDC1 downregulation; shDEPDC1+sheL31: DEPDC1 downregulation and eL31 downregulation. The data are expressed as mean ± SD. ** *P* < 0.01, *** *P* < 0.001.

Table S1. Antibodies used in western blotting and IHC.

| Primary antibodies | Dilution in WB | Source species | Company | Catalog No. |
| --- | --- | --- | --- | --- |
| eL31 | 1:500 | Rabbit | abcam | ab103991 |
| CDCA5 | 1:1000 | Rabbit | abcam | ab192237 |
| DEPDC1 | 1:1000 | Rabbit | abcam | ab197246 |
| MCM8 | 1:1000 | Rabbit | abcam | ab191914 |
| NEK2 | 1:1000 | Rabbit | abcam | ab115731 |
| Akt | 1:1000 | Rabbit | CST | 4685 |
| P-Akt | 1:500 | Rabbit | R&D | AF887-sp |
| CCND1 | 1:2000 | Rabbit | CST | 2978 |
| CDK6 | 1:1000 | Rabbit | abcam | ab151247 |
| PIK3CA | 1:1000 | Rabbit | abcam | ab40776 |
| DEPDC1 | 1:10/1:500 | Rabbit | sigma | SAB1303485 |
| GAPDH | 1:3000 | Rabbit | Bioworld | AP0063 |
| Secondary antibody | Dilution |  | Company | Catalog No. |
| Goat Anti-Rabbit | 1:3000 |  | Beyotime | A0208 |

| Primary antibodies | Dilution in IHC | Source species | Company | Catalog No. |
| --- | --- | --- | --- | --- |
| eL31 | 1:100 | Rabbit | abcam | ab103991 |
| DEPDC1  Ki67 | 1:100  1:200 | Rabbit  Rabbit | Sigma  abcam | SAB1303485  Ab16667 |
| Secondary antibody | Dilution |  | Company | Catalog No. |
| Goat Anti-Rabbit | 1:400 |  | abcam | A6721 |

Table S2. Primers used in qRT-PCR.

| **Gene** | **Forward primer sequence (5′-3′)** | **Reverse primer sequence (5′-3′)** |
| --- | --- | --- |
| eL31 | GCTCAACAAAGCTGTCTGGG | TTATGGGCTCTTGGCGACT |
| AARS | CACTGCTTGGCTCCTCTATGA | CTCCCTTGCCCTGTGATTT |
| BARD1 | ATCCTCAGTAGAAAGCCCAAGC | TCAGAATCGGGTCTCGCATG |
| BLM | ACGGATTTTGTTCCACCTTCTC | CAGCTCCTGCATACTCATTTTC |
| BRD7 | ACTGATTTTATTGCTCCTGG | TGAAGTTATCCTTTAGTTCTTCT |
| CDCA5 | CCGAGCATCCTCCCTGAAAT | CATGGGCCACGATCCTCTTTA |
| DEPDC1 | CTGAAGTGACCCGCAAACAAA | CTGGTGGGAGATCATTCCATTC |
| FANCA | GTTTGAGCATACGGGGAACA | TGGCAGTAGGTGGAGTACAGAG |
| FANCB | TGATAGCTTGGTTGTTGGAGTG | AAGTATGGTGCTGGGAAAGGA |
| FANCD2 | GGCTTGACAGAGTTGTGGATG | TAGGATCTCAGGTAGGCTGGTG |
| HMGB2 | TCGCTATGACAGGGAGATGAA | AGGAAGAAGGCAGATGGTGG |
| MARS | CATTACTGCAAGATCCCGCC | TTTGGAGGTAAGGCCGGAGA |
| MCM8 | ATGGCTTTTCTTTGTGCTGC | CCAGTCCATCGTAACTGTGAGA |
| NEK2 | GAAGGAATGCCACAGACGAAGT | CAAGCAGCCCAATGACCAGATA |
| OIP5 | CTTCCTAGTTGGCATTGAAGGT | CAGCATGGGTAGAATACAGATGGA |
| PLK1 | AGAGGAGGAAAGCCCTGACT | TAACTCGGTTTCGGTGCAGG |
| RFC4 | TCAGGAAGTCGCTCAGATGG | CAGAATCGGGTGGTTTTCG |
| SKA1 | TCCCATTTGCCTCAAGTAACAG | TCCCCAACACACAGACTCCA |
| TARS | AAGATAGCAAGCCAATTAAAGTCAC | CAGGCAATTTGATATGGTGTAG |
| TOPBP1 | TGAATCTAACATCGCAAACATC | CACAGCATAATCCGCAACAG |
| TRIB3 | AGCGGTTGGAGTTGGATGA | TTGCACGATCTGGAGCAGTAG |
| GAPDH | TGACTTCAACAGCGACACCCA | CACCCTGTTGCTGTAGCCAAA |
